# The reliability and validity of a screening scale for online gaming disorder among Chinese adolescents and young adults

**DOI:** 10.1186/s12888-021-03678-1

**Published:** 2022-01-10

**Authors:** Xuechan Lyu, Tianzhen Chen, Zhe Wang, Jing Lu, Chenyi Ma, Haoye Tan, Runji Li, Peiyan Wang, Limin Ma, Hongwei Li, Shuqin Hui, Wenli Zhao, Jiang Long, Na Zhong, Min Zhao

**Affiliations:** 1 Shanghai Mental Health Center, Shanghai Jiao Tong University School of Medicine, 600 Wan Ping Nan Road, Shanghai, 200030 China; 2 University of California Los Angeles, Los Angeles, USA; 3 Lulong Vocational and Technical Education Center, Qinhuangdaoa, Hebei China; 4 Shanghai Key Laboratory of Psychotic Disorders, Shanghai, China; 5 CAS Center for Excellence in Brain Science and Intelligence Technology (CEBSIT), Chinese Academy of Sciences, Shanghai, China

**Keywords:** Online gaming disorder, Screening, Chinese

## Abstract

**Background:**

In recent years, there have been frequent reports of gaming disorder in China, with more focus on young people. We developed and psychometrically tested a Gaming Disorder screening scale (i.e., Gaming Disorder Screening Scale - GDSS) for Chinese adolescents and young adults, based on the existing scales and diagnostic criteria, but also considering the development status of China.

**Methods:**

For testing content and criterion validity, 1747 participants competed the GDSS and the Internet Addiction Test (IAT). After 15 days, 400 participants were retested with the scales for to assess test-retest reliability. Besides, 200 game players were interviewed for a diagnosis of gaming disorder.

**Results:**

The Cronbach’s alpha coefficient on the GDSS was 0.93. The test-retest coefficient of 0.79. Principal components analysis identified three factors accounting for 62.4% of the variance; behavior, functioning, cognition and emotion. Confirmatory factor analysis showed a good model fit to the data (χ^2^ /df = 5.581; RMSEA =0.074; TLI = 0.916, CFI = 0.928). The overall model fit was significantly good in the measurement invariance tested across genders and different age groups. Based on the clinical interview, the screening cut-off point was determined to be ≥47 (sensitivity 41.4%, specificity 82.3%).

**Conclusions:**

The GDSS demonstrated good reliability and validity aspects for screening online gaming disorder among Chinese adolescents and young adults.

**Supplementary Information:**

The online version contains supplementary material available at 10.1186/s12888-021-03678-1.

## Introduction

Gaming disorder is defined as a pattern of gaming behavior characterized by impaired control over gaming, increasing priority given to gaming over other activities to the extent that gaming takes precedence over other interests and daily activities, and continuation or escalation of gaming despite the occurrence of negative consequences [1]. The American Psychiatric Association [2] has proposed Internet gaming disorder (IGD) as a potential addictive disorder in the fifth edition of the Diagnostic and Statistical Manual of Mental Disorder (DSM-5), and more recently, the World Health Organization has included gaming disorder (GD) (predominantly online or offline) in the International Classification of Diseases 11th Revision (ICD-11) [1]. In recent years, there have been frequent reports of gaming disorder in China, with more focus on young people. According to a joint report released by the government-affiliated China Internet Network Information Center and the Chinese Communist Youth League, Chinese juvenile Internet users reached 183 million, and the Internet penetration rate of juveniles reached 94.9% in 2020 [3]. The data showed that 62.5% of the underage Internet users often play online games and presented an increasing trend year by year. A study based on 10 provinces in China showed that 79.3% of Chinese adolescents played online games, while 3.2% showed behavior addiction [4]. An analysis of 36 representative survey studies in China noted that the Chinese literature may be jeopardized by inappropriate identification of problematic online gaming because there is no consensus or gold standard regarding its diagnostic criteria [5].

Prior to the release of the DSM-5, there was no gold standard of Internet gaming disorder classification, so the prevalence estimate varied widely due to difference in assessment methods and surveyed population (0.2% ~ 46%) [6]. In problematic gaming and gaming disorder screening, a common practice was to use tools adapted from Young’s Internet Addiction Test [7] or scales for other addictive behaviors [8–11]. In recent years, screening scales for gaming disorder have tended to list the DSM-5 criteria directly [12], the ICD-11 [13] or adapted ones [14, 15]. In the recent King’s systematic review, AICA-S gaming, GAS-7, IGDT-10, IGDS9-SF, and Lemmens IGD-9, had greater evidential support for their psychometric properties, but there was no markedly superior tool with distinct practical and/or psychometric advantages [16]. There is still some uncertainty or lack of agreement among researchers about the best methods for screening and evaluation. For now, most of the scales reported in China were adapted from foreign scales, and their applicability might be affected by language/cultural influences [17–19]. And there are no Chinese screening tools based on ICD-11 diagnostic criteria. The development of scales based on new diagnostic criteria is meaningful for each country, besides its international importance, which will optimize our methods of screening potential populations of disease and improves efficiency.

In order to facilitate future research on gaming disorder in China, while there was a lack of international general scales and the introduction of unaccredited scales often has some linguistic and cultural problems, we integrated the existing scales and diagnostic criteria, combined with the development status of China, and developed the Gaming Disorder Screening Scale (GDSS), an online gaming disorder screening scale for Chinese population. We tested the psychometric properties of GDSS and identified a screening cut-off score in school adolescents and young adults.

## Methods

### Participants

Using convenience sampling, 2140 middle school students were enrolled from two middle schools in Qinhuangdao City, Hebei Province, China from 2018 to 2019. One middle school was a general public high school, and the other was secondary vocational and technical school, which covered as many different types of teenagers as possible. Inclusion criteria were: age over 12 years old; with normal or corrected-to-normal sight and hearings; voluntary participation in the study; the subjects themselves and at least one of their parents/monitors signed the informed consent. Exclusion criteria were: not playing games; mental disorder or severe physical disease; severe cognitive impairment, or any inability to fill out questionnaires. The participation was voluntary without any monetary compensation.

### Measures

#### Gaming Disorder Screening Scale (GDSS, Additional file 1)

The GDSS was designed based on the DSM-5 and ICD-11 diagnostic criteria and the cognitive psychology of Internet gaming disorder [20]. The scale’s original version contained 25 items, covered DSM-5 and ICD-11 symptoms as much as possible. After the discussion of experts and a pilot study of 250 participants, the final version was formed. It consists of 18 Likert-scaled items with 4 choices (1, never;2, sometimes;3, ofen;4, always) and has a theoretical value range of 18–72. Ratings represent an average score for the past year. The items contain all of the three criteria of ICD-11(item 1,3,5,8,9,10,13,16,17), and eight symptoms of DSM-5 without ‘Escapism’ (item 1–11, and 14–18). The ‘escapism’ criteria had relatively lower diagnostic accuracy in some papers [21, 22]. Item 12 referred to one of the GD specific cognitions “maladaptive and inflexible rules about gaming” [20]. Moreover, the ‘Tolerance’ criteria were always relevant to the question ‘I feel a continued need to play more and more Internet games’ in the previous scales. We modified the question in the way of ‘I need to keep breaking records (or passing) to get the excitement I want (or want to be a master or a strong player in the game)’, which is more in line with the situation of gaming cognition in Chinese population.

#### Internet Addiction Test (IAT)

The IAT consists of 20 Likert-scaled items (0, not applicable to your life; 1, rarely; 2, occasionally; 3, frequently; 4, often; 5, always) and has a theoretical value range of 0–100 [23]. The IAT total score ranges, with the higher the score representing the higher level of severity of Internet compulsivity and addiction. Total scores that range from 0 to 30 points are considered to reflect a normal level of Internet usage; scores of 31 to 49 indicate the presence of mild risk of Internet addiction; 50 to 79 reflect the presence of a moderate risk level; and scores of 80 to 100 indicate high risk of Internet addiction. In the clinical interview of this study, 3 groups of participants divided by scores of IAT were high risk group (> 50), medium risk group (30 ~ 50) and low risk group (< 30).

#### Clinical interview

A clinician’s diagnosis of gaming disorder was based on the ICD-11 criteria. Based on the IAT scores, there were 120 participants who scored above 50 points and accepted to be interviewed. In order to include some lower risk participants into the clinical interview, we selected 60 middle-risk participants (from 811 participants) and 20 low-risk participants (from 816 participants) by stratified random sampling at a ratio of 6: 3: 1. A total of 200 participants were invited to join the face-to-face interview. Each gaming player was joint-interviewed and independently diagnosed by 2 psychiatrists. One psychiatrist acted as a major interviewer and the other as an observer. At the end of the interview, the observer was allowed to ask additional questions. Each psychiatrist drew a diagnosis independently according to ICD-11 diagnostic guidelines. The two psychiatrists ‘identical diagnoses were indeed the final diagnosis. The participating psychiatrists had more than 3 years of work experience and the ability and qualification to diagnose mental disorders independently. A total of seven psychiatrists participated in the clinical interview, all of whom received training of the diagnostic criteria of Gaming Disorder in ICD-11.

### Procedure

Of 2140 participants enrolled in this study, 1747 provided valid data. Each participant was assisted to finish the IAT and the GDSS in their classroom by our trained research assistants. The game activities of all the subjects were online. Participants were between 13 and 23 years old, and average age was 15.59 years (SD = 1.60). One thousand thirteen participants (58%) were male. Two hundred subjects who met the risk criteria for IAT scores were invited to join in the clinical interview within 4 weeks, and 193 provided valid data. This sample of gamers were between 14 and 20 years old, average age was 16.51 years (SD = 1.04), and consisted of 86% males (*n* = 166). Twenty-nine participants were diagnosed with gaming disorder (26 males and 3 females) and 17 participants were diagnosed with hazardous gaming (14 males and 3 females). After 15 days, a random sample of 400 subjects was retested on the scales.

### Statistical analysis

Cronbach’s alpha coefficient was used to evaluate the internal consistency of the GDSS. Pearson correlation was used to evaluate test-retest reliability and convergent validity between the GDSS and the IAT sum scores. The logistic regression was used to evaluate the correlation between the GDSS and the clinical diagnosis. The data was split randomly and on the first to do principal components analysis (PCA) and on the second to do confirmatory factor analysis (CFA). PCA was conducted using an oblique factor rotation. The number of factors was based on an examination of eigenvalues greater than 1. In order to determine whether the model had a good fit, we used TLI and CFI values ≥0.90 and RMSEA ≤0.08 as cut-of values CFA. With the whole sample(*n* = 1747), a multi-group analysis was performed to assess whether the model from the CFA were invariant across gender and age. Gender was divided into male (*n* = 1013) or female (*n* = 734), age was divided into the below (*n* = 877) or above (*n* = 855) the mean of 15.59 years, and unknown age data were excluded (*n* = 15). The following types of invariances were considered: configural invariance (model without constraints), metric invariance (equality of factorial weights), scalar invariance (factorial weights and covariance equals) and residual invariance (factorial weights, covariance and equal measure errors). Given the fact that Chi-square test is strongly influenced by sample size [24], we adopted to observe the change of CFI between nested models in order to assess measurement invariance. Change in RMSEA less than 0.015 is considered acceptable. To determine a threshold for the GDSS, we computed the sensitivity and specificity for different GDSS cut-off points referring to the diagnosis of gaming disorder through ICD-11. The cutoff point with the highest youden’s index was determined as the optimal cutoff point. The significance level was *P* < 0.05. All analyses were developed using SPSS v. 20.0 and Amos v. 22.0.

### Ethics

The ethical approval for this study (ref. no.: 20189–73) was obtained from the Ethics Committee of Shanghai Mental Health Center. All study procedures were conducted in accordance with the Declaration of Helsinki. All participants provided informed consent for their participation, while parents’ permission was also obtained for those less than 18 years of age.

## Results

For the GDSS, the data revealed a mean sum score of 34.17 (SD = 10.32) and a range of 19–72.

### Internal consistency and test-retest reliability

Cronbach’s alpha on the GDSS was 0.93, and Guttman split-half coefficient was 0.87. Correlation efficiencies between each item and total score corrected for that item ranged from 0.55–0.74. The test-retest coefficient of 0.79 (*p* < 0.01).

### Structural validity

The KMO sample suitability test and Bartlett spherical test were performed on 18 items of the GDSS, and the results showed that KMO =0.95, Bartlett test *P* < 0.01, indicating that the data was suitable for factor analysis. Three factors were identified with eigenvalues greater than 1 (Table 1). All three factors accounted for 62.4% of the variance. The first factor explained 44.57% of the variance and contained 6 items (1–3, 5, 7, and 9), which represented behaviors related to gaming. The second factor explained 10.06% of the variance and contained 5 items (8, 10, 13, 16, and 17), which represented as functioning. The third factor explained 7.76% of the variance and contained 7 items (4, 6, 11, 12, 14, 15, and 18), which represented as cognition and emotion.

**Table 1:** Rotating Component Matrix

Item	Behavior	Functioning	Cognition and Emotion
5. I’ve tried to reduce the amount of time I spend playing online games, but it doesn’t work	.86		
3. Because of playing online games, I am less interested in other activities than before (e.g. meeting friends offline, spending time with my parents)	.84		
9. I feel like I can’t control the time I spend playing online games	.82		
1.My interests have changed as a result of playing online games (e.g., I used to love sports, but now I love online games related activities)	.79		
7. I actually spend more time on online games than I promise others	.76		
2. I will lie to my family or teachers to cover up the real time I spend playing online games	.74		
10. I risk losing important friendships or family relationships to play online games (e.g., reducing contact with friends or being ostracized by friends for frequent online games, conflicting with parents over excessive use of online games)		.88	
17. Because of my frequent online games, real-life friends have gradually reduced contact with me		.86	
8. Because I play online games frequently, I get into trouble at school (e.g. declining academic performance and lack of success in class)		.84	
13. I’ll ignore what I’m supposed to do because I’m online (e.g., I was planning to do my homework, but I’m postponing it because I’m playing online games)		.83	
16. My family will complain to me because I have played online games longer than they expected		.80	
11. I get impatient and even angry when someone disturbs me to play online games			.89
4. I need to keep breaking records (or passing) to get the excitement I want (or want to be a master or a strong player in the game)			.84
12. When I didn’t break the record (or didn’t pass), I thought I’d make it next time			.72
14. When I can’t play online games, I get irritable or unhappy			.71
18. When I can’t play online games, I get anxious and pressured			.63
15. I’ll plan the time or content of the next game			.59
6. Even when I’m not playing online games, game-related content comes to my mind			.52

**Fig. 1:**
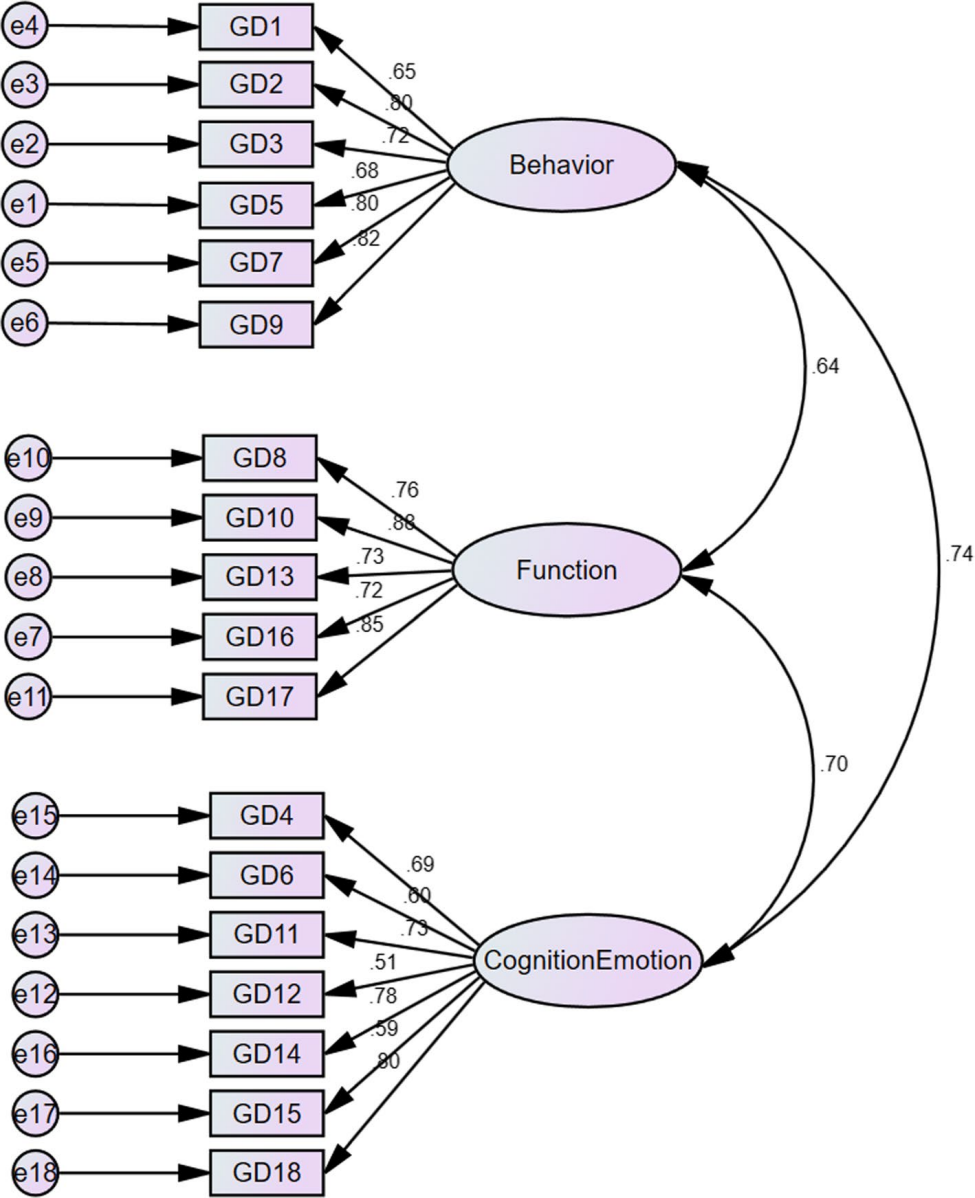
Standardized individual parameters (covariance factors, factorial weights and measurement errors)

Secondly, we did the CFA with the other half of data (*n* = 843) to confirm the factors found in PCA, which showed a good model (χ^2^ /df = 5.581; RMSEA =0.074; TLI = 0.916, CFI = 0.928). All factor loadings were satisfactorily high (0.51–0.88; Fig. 1).

With the whole sample(*n* = 1747), the overall model fit was significantly well in the measurement invariance across genders and across different age groups (Table 2). Both gender and both age groups presented good fit indices (ΔRMSEA < 0.015).

**Table 2:** Multi-group analysis of fit indices by gender and age groups

Model	χ2/df	p	TLI	CFI	RMSEA	Δ-RMSEA	Δ-CFI
Across gender group							
Male	5.98	<.001	0.91	0.93	0.07	/	/
Female	4.70	<.001	0.93	0.94	0.07	/	/
Configural invariance	5.34	<.001	0.92	0.93	0.05	/	/
Metric invariance	5.14	<.001	0.92	0.93	0.05	−0.001	0
Scalar invariance	5.22	<.001	0.92	0.93	0.05	−0.001	−0.007
Residual invariance	5.60	<.001	0.91	0.92	0.05	0.001	−0.019
Across age group							
Below mean age	7.54	<.001	0.91	0.92	0.08	/	/
Above mean age	4.05	<.001	0.90	0.91	0.06	/	/
Configural invariance	5.79	<.001	0.89	0.92	0.05	/	/
Metric invariance	5.65	<.001	0.90	0.92	0.05	−0.001	−0.003
Scalar invariance	8.23	<.001	0.84	0.86	0.07	0.012	−0.061
Residual invariance	8.64	<.001	0.83	0.84	0.07	0.013	−0.078

*χ2* chi-squared, *df* degrees of freedom, *CFI* comparative fit index, *TLI* Tucker-Lewis Index, *RMSEA* root-mean-square error of approximation, *ΔCFI* differences in the value of the Comparative Fit Index, *ΔRMSEA* differences in the value of the root-mean-square error of approximation

### Criterion-related validity

The sum score of the IAT was analyzed with the sum score of GDSS, and the results showed that the correlation coefficient was 0.53, *p* < 0.01, which meant GDSS was positively related to the IAT. Secondly, the correlation between clinical diagnosis of GD and the sum score of GDSS was analyzed through logistic regression. The result showed that the GDSS score was positively correlated with the clinical diagnosis of GD, and the probability of diagnosis increased by 4% for every point higher in the GDSS score (*n* = 193, OR = 1.04, 95% CI: 1.01–1.08).

### Cut-off values of GDSS

**Fig. 2:**
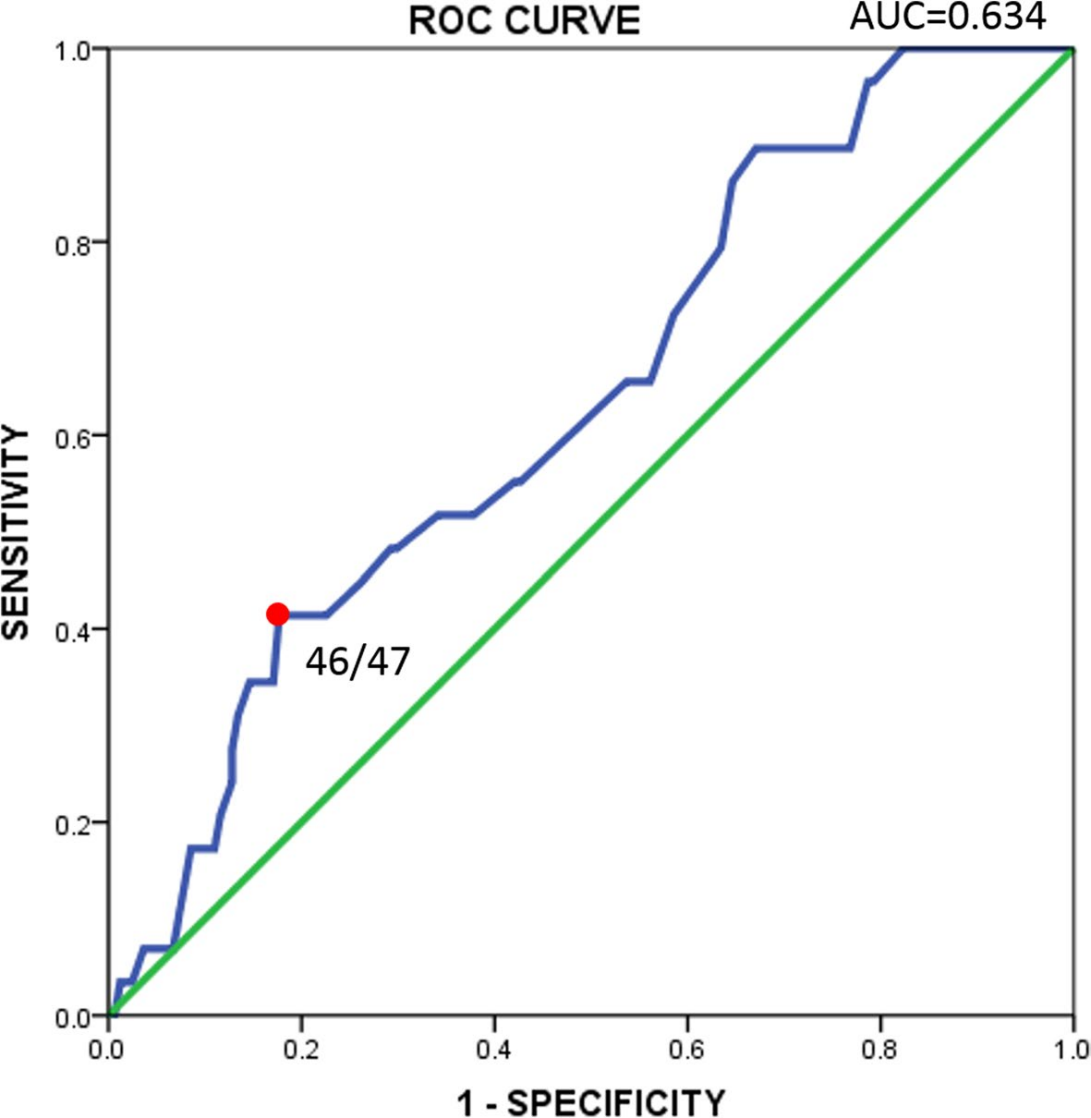
Results of the area under the receiver operating characteristic curve analysis for the GDSS for classifying gaming disorder (according to the clinical diagnosis)

The screening efficacy was operationalized as AUC from the ROC analysis. We compared the GDSS score with clinical diagnosis results to compute a screening cut-off point. We adopted the gaming disorder diagnosis based on ICD-11 as gold standard to conduct ROC analysis on the GDSS and found a moderate efficacy (*n* = 193, AUC = 0.63, 95% CI 0.53–0.74) (Fig. 2). Cut-off point for GDSS to present high risk of gaming disorder was above or equal 47 (sensitivity 41.4%, specificity 82.3%).

## Discussion

In this study, we reported the psychometric properties of the 18-item GDSS, which was the first gaming disorder screening tool based on DSM-5 and ICD-11 developed in Chinese. The development of screening scales based on new diagnostic criteria and new situation will optimize our approach to screening potential populations of disease and improve efficiency, which is very meaningful. At present, there have been some scales developed based on DSM-5 or ICD-11 diagnostic criteria for gaming disorders, but it was still lacked in China. Any adapted English scale contains nuance in its vocabulary, and these biases can subconsciously influence people’s judgment. A cross-culture study on IGD scale has approved language or cultural influences on some symptoms, such as pre-occupation with games [13]. Therefore, we developed such a scale in order to improve the research methods of gaming disorder by more Chinese researchers.

In terms of the coverage of diagnostic items, the GDSS contains all of the three criteria of ICD-11 and eight symptoms of DSM-5 without ‘Escapism’. King et al. [16] summarized the existing gaming disorder scales in 2020, including a total of 32 scales. The GDSS was different from the above 32 scales. The A-EQ [9], POGU [25], POGQ [26], GAIT [27] scales didn’t include “Escape”, but covered less diagnostic items. In addition to the diagnostic criteria, we also referenced to the cognitive psychology of Internet gaming disorder [20] and incorporated it into GDSS, such as item 12. In order to better adapt to the characteristics of adolescents, in terms of functional damage, we covered social and educational damage, excluding work and finance.

The overall scale of the GDSS displayed high internal consistency and good stability. Analysis of the scale items and the rest of the scale correlation coefficient showed that no items were with a low overall relevance to the scale. Criterion validity of GDSS was proved by positive correlations with clinical diagnosis and IAT scores. IAT was a commonly used instrument of internet addiction, not specially for internet gaming disorder. Our research started in 2018, when there was no recognized authoritative gaming disorder scale in China. The Chinese version of IAT was a mature scale with good reliability and validity, and there was a lot of research evidence. Many symptoms of IGD overlap with symptoms of Internet addiction, as evidenced in other previous study [28]. GDSS score was significantly correlated with clinical diagnosis, suggesting that the higher the score, the stronger the risk of game disorder. We believed that GDSS could be a good assistant tool for Chinese clinical work.

In the structural validity test three factors were identified, confirmed by CFA. In the first factor, the 6 items referred to the reduction of other activities in life due to excessive play and the deception caused by excessive play. This dimension was named ‘behavior’. A study based on the DSM-5 showed that ‘give up other activities’, ‘tolerance’ and ‘withdrawal’ were key importance for identifying IGD [29]. The second factor was named ‘functioning’ as these 5 items indicated academic problems caused by excessive play, as well as parent-child and peer relationship problems. However, some studies found that“negative consequences” was the least endorsed criteria for all gamers [29, 30]. However, ‘continuation of gaming despite the occurrence of negative consequence’ is emphasized as a third diagnostic criterion in ICD-11. The 7 items of the third factor concerned about continuous desire to play, thinking back to the game scene, imagination on the next play, and the feeling changes due to playing games. This factor was named ‘cognition and emotion’. In theory, it should be two dimensions, but factor analysis does not separate them, probably because cognition is closely related to emotions. In the measurement invariance, the GDSS scale was invariant across genders and different age groups, which indicates that our scale could be used for cross-gender comparisons, and relatively independent of the age effects.

The GDSS displayed a moderate overall diagnosis efficacy in detecting ICD-11-proposed gaming disorder cases (AUC = 0.63), presumably due to a small number of confirmed cases (29 subjects). By setting ≥47 as the screening cut-off point, we found the efficacy of the GDSS (41.4% sensitivity and 82.3% specificity) was optimized in identifying probable gaming disorder cases among Chinese adolescent people. The efficacy proposed by Chen et al. [31] (81.7% sensitivity and 85.4% specificity) for the Chinese Internet Gaming Disorder Checklist (C-IGDC) was comparable to what was proposed by Ko et al. [32](83.9% sensitivity and 76.7% specificity) for the Chen Internet Addiction Scale (CIAS), as both are screening tools of Internet-related addiction developed in Chinese samples. Their results seemed to be better, but the references in their case were other scales, not clinical diagnosis as a gold standard, as we did. There was also a study that used clinical interviews to determine cut-off points and achieved high diagnostic efficiency (AUC = 0.81), but this study had relatively small sample size [17].

The present study also had some limitations and unsolved issues. Firstly, the subjects of this study were all middle school students in Qinhuangdao City, Hebei Province, China, which limited the extent of generalizability of our findings on the psychometric soundness of the GDSS to broader population. However, it is hoped that future studies can confirm the findings presented here across the country. Secondly, the number of subjects in our study who participated in clinical interviews was relatively small that the validity of the current proposed screening cut-off point needs to be further validated. We call for further validation of the GDSS to be conducted with a more general gamer sample across different demographic and player groups. Thirdly, we didn’t get the information about play time, which is important to understand the usage of the game. Finally, probable self-report bias is inevitable when using survey methods, and future research should consider collecting more behavioral data for comparison.

## Conclusion

The GDSS is the only gaming disorder screening tool based on the ICD-11 and DSM-5 specifically in Chinese. The results of the present study supported the reliability and validity of GDSS for screening gaming disorder cases among Chinese adolescents and young adults, although the diagnostic thresholds need to be further studied in additional samples.

## Supplementary Information

**Additional file 1.**

## Data Availability

The datasets used and/or analyzed during the current study are available from the corresponding author on reasonable request.
