# Supplementary material for: The reliability and validity of a screening scale for online gaming disorder among Chinese adolescents and young adults
Source: BMC Psychiatry. 2022 Jan 10;22:28. doi: 10.1186/s12888-021-03678-1 (PMC8751106; doi:10.1186/s12888-021-03678-1)
Supplement: Supplementary file 1 — Additional file 1. [file 12888_2021_3678_MOESM1_ESM.doc]

**Appendix 1**

**Gaming Disorder Screening Scale (GDSS)**

The following 18 items are designed to help you understand how online games affect your studies and aspects of your life. Please read them carefully and answer them as you see fit, there is no right or wrong answer. Please base your answers on what you have done in the last 12 months.

|  | never | sometimes | often | always |
| --- | --- | --- | --- | --- |
| 1. My interests have changed as a result of playing online games (e.g., I used to love sports, but now I love online games related activities) | **1** | **2** | **3** | **4** |
| 2. I will lie to my family or teachers to cover up the real time I spend playing online games | **1** | **2** | **3** | **4** |
| 3. Because of playing online games, I am less interested in other activities than before (e.g. meeting friends offline, spending time with my parents) | **1** | **2** | **3** | **4** |
| 4. I need to keep breaking records (or passing) to get the excitement I want (or want to be a master or a strong player in the game) | **1** | **2** | **3** | **4** |
| 5. I've tried to reduce the amount of time I spend playing online games, but it doesn't work | **1** | **2** | **3** | **4** |
| 6. Even when I'm not playing online games, game-related content comes to my mind | **1** | **2** | **3** | **4** |
| 7. I actually spend more time on online games than I promise others | **1** | **2** | **3** | **4** |
| 8. Because I play online games frequently, I get into trouble at school (e.g. declining academic performance and lack of success in class) | **1** | **2** | **3** | **4** |
| 9. I feel like I can't control the time I spend playing online games | **1** | **2** | **3** | **4** |
| 10. I risk losing important friendships or family relationships to play online games (e.g., reducing contact with friends or being ostracized by friends for frequent online games, conflicting with parents over excessive use of online games) | **1** | **2** | **3** | **4** |
| 11. I get impatient and even angry when someone disturbs me to play online games | **1** | **2** | **3** | **4** |
| 12. When I didn't break the record (or didn't pass), I thought I'd make it next time | **1** | **2** | **3** | **4** |
| 13. I'll ignore what I'm supposed to do because I'm online (e.g., I was planning to do my homework, but I'm postponing it because I'm playing online games) | **1** | **2** | **3** | **4** |
| 14. When I can't play online games, I get irritable or unhappy | **1** | **2** | **3** | **4** |
| 15. I'll plan the time or content of the next game | **1** | **2** | **3** | **4** |
| 16. My family will complain to me because I have played online games longer than they expected | **1** | **2** | **3** | **4** |
| 17. Because of my frequent online games, real-life friends have gradually reduced contact with me | **1** | **2** | **3** | **4** |
| 18. When I can't play online games, I get anxious and pressured | **1** | **2** | **3** | **4** |

| Behavior | Cognition and emotion | Function |
| --- | --- | --- |
| **1, 2, 3, 5, 7, 9** | **4, 6, 11, 12, 14, 15, 18** | **8, 10, 13, 16, 17** |
